# Endurance Training with or without Glucose-Fructose Ingestion: Effects on Lactate Metabolism Assessed in a Randomized Clinical Trial on Sedentary Men

**DOI:** 10.3390/nu9040411

**Published:** 2017-04-20

**Authors:** Robin Rosset, Virgile Lecoultre, Léonie Egli, Jérémy Cros, Valentine Rey, Nathalie Stefanoni, Valérie Sauvinet, Martine Laville, Philippe Schneiter, Luc Tappy

**Affiliations:** 1 Department of Physiology, University of Lausanne, CH-1005 Lausanne, Switzerland; Robin.Rosset@unil.ch (R.R.); Virgile.Lecoultre@hibroye.ch (V.L.); Leonie.Egli@rdsg.nestle.com (L.E.); Jeremy.Cros@unil.ch (J.C.); Valentine.Rey@unil.ch (V.R.); Nathalie.Stefanoni@unil.ch (N.S.); Philippe.Schneiter@unil.ch (P.S.); 2 Centre for Research in Human Nutrition Rhône-Alpes and European Centre of Nutrition for Health, Lyon 1 University, INSERM, Hospices Civils de Lyon, F-69310 Pierre Bénite, France; Valerie.Sauvinet@chu-lyon.fr (V.S.); Martine.Laville@chu-lyon.fr (M.L.)

**Keywords:** glucose, fructose, lactate, lactate metabolism, substrate oxidation, carbohydrate, exercise

## Abstract

Glucose-fructose ingestion increases glucose and lactate oxidation during exercise. We hypothesized that training with glucose-fructose would induce key adaptations in lactate metabolism. Two groups of eight sedentary males were endurance-trained for three weeks while ingesting either glucose-fructose (GF) or water (C). Effects of glucose-fructose on lactate appearance, oxidation, and clearance were measured at rest and during exercise, pre-training, and post-training. Pre-training, resting lactate appearance was 3.6 ± 0.5 vs. 3.6 ± 0.4 mg·kg^−1^·min^−1^ in GF and C, and was increased to 11.2 ± 1.4 vs. 8.8 ± 0.7 mg·kg^−1^·min^−1^ by exercise (Exercise: *p* < 0.01). Lactate oxidation represented 20.6 ± 1.0% and 17.5 ± 1.7% of lactate appearance at rest, and 86.3 ± 3.8% and 86.8 ± 6.6% during exercise (Exercise: *p* < 0.01) in GF and C, respectively. Training with GF increased resting lactate appearance and oxidation (Training × Intervention: both *p* < 0.05), but not during exercise (Training × Intervention: both *p* > 0.05). Training with GF and C had similar effects to increase lactate clearance during exercise (+15.5 ± 9.2 and +10.1 ± 5.9 mL·kg^−1^·min^−1^; Training: *p* < 0.01; Training × Intervention: *p* = 0.97). The findings of this study show that in sedentary participants, glucose-fructose ingestion leads to high systemic lactate appearance, most of which is disposed non-oxidatively at rest and is oxidized during exercise. Training with or without glucose-fructose increases lactate clearance, without altering lactate appearance and oxidation during exercise.

## 1. Introduction

During moderate and high intensity exercise, muscle energy needs are essentially met by carbohydrate oxidation [1] and muscle performance is dependent on both muscle glycogen and plasma glucose concentrations [2,3,4]. Plasma glucose entry into skeletal muscle is activated by contraction [5] and depends on systemic glucose appearance, i.e., the sum of endogenous glucose production (hepatic glycogen breakdown and gluconeogenesis) and gut glucose absorption [1,6]. Accordingly, whole-body and muscle glucose oxidation can be enhanced by glucose ingestion that increases plasma glucose appearance and muscle glucose uptake [6]. A portion of glucose is actually made indirectly available through glycolytic lactate production in some muscle fibers, followed by lactate uptake and oxidation in other fibers [7]. These lactate “shuttles” may possibly increase muscle energy substrates provision when glucose transport and/or glycolytic capacity is saturated. Endurance training has also been well-established to increase the expression of proteins involved in lactate transport and metabolism [8], suggesting that lactate can be a major energy substrate for the trained muscle [7,9]. Alternatively, lactate can also be recycled into glucose (gluconeogenesis), with recent indications that this can be altered in trained subjects [10,11].

The oxidation rate of exogenous glucose increases dose-dependently up to ≈1 g·min^−1^, but then plateaus at higher glucose ingestion rates [4]. Co-ingestion of glucose and fructose can further increase exogenous [12] and net carbohydrate oxidations [13], and can also improve exercise performance [14]. This has been attributed to glucose and fructose being absorbed through distinct transporter proteins in the apical membrane of enterocytes, thus allowing for a higher rate of total gut carbohydrate absorption [4]. In addition, a substantial portion of ingested fructose is converted into lactate in the liver, to increase plasma lactate concentration and lactate delivery as an energy substrate to working muscle [15,16].

Fructose absorption is poor when it is ingested alone, but is markedly enhanced by glucose co-ingestion [17]. Furthermore, gut fructose transport is potently induced by fructose itself, and fructose absorption increases within a few days upon chronic fructose ingestion [9]. One may therefore suspect that the beneficial effects of ingesting glucose and fructose mixtures during exercise may depend on their chronic use during training. In addition, hyperlactatemia is thought to be instrumental in training-induced increase in lactate clearance by the stimulation of muscle lactate transporters [9]. We therefore postulated that the combined, repeated effects of exercise and glucose-fructose ingestion may significantly impact training-induced adaptation of muscle lactate metabolism. To assess this hypothesis, we enrolled two groups of healthy sedentary males in a 3-week training program during which they consumed glucose-fructose drinks (GF intervention) or plain water as a control (C intervention) during training sessions.

## 2. Materials and Methods

### 2.1. Participants

Sixteen healthy young males (mean ± standard error (SEM) age: 25 ± 1 years; weight: 73.2 ± 2.0 kg; body mass index: 22.9 ± 0.4 kg·m^−2^) completed this study. One additional volunteer dropped out prior to the interventions and was hence removed from all analyzes. At inclusion, all participants were sedentary and low-sugar consumers (exercise: <1 h·week^−1^ and sugar intake: <60 g·day^−1^) and were asked to maintain their lifestyle with the exception of the supervised exercise sessions prescribed in the study protocol. They were fully informed of the nature and risks involved by the procedures, in accordance with the 1983 revision of the Declaration of Helsinki. All experiments were performed at the Clinical Research Center, Lausanne, Switzerland, after approbation by the local ethics committee. This study was registered at ClinicalTrials.gov database as NCT01610986.

### 2.2. Study Design

**Figure 1:**
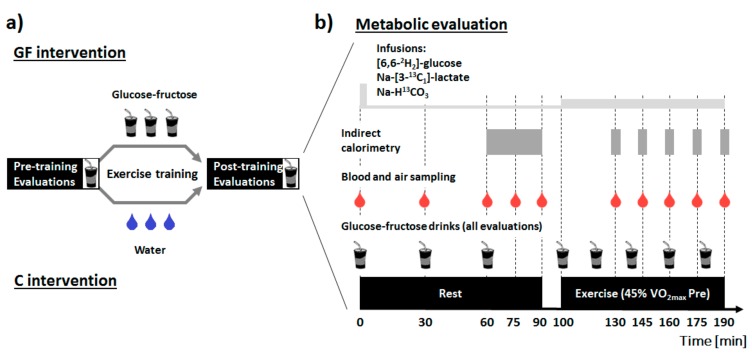
Study design (**a**) and description of the metabolic evaluations (**b**). Drinks containing 19 g glucose and 12 g fructose were administered at time 0, 30 and 60 min at rest, and at 20 min intervals during exercise. Primed-continuous infusions of (6,6-^2^H_2_)-d-(+)-glucose and Na-(3-^13^C_1_)-l-(+)-lactate were started at time 0, and resting measurements were obtained after 60 min equilibration. Continuous infusion rates were upgraded at the beginning of exercise at time 100 min (see methods for further details). GF: intervention in which glucose-fructose drinks were provided during training sessions; C: control intervention in which plain water was provided during training sessions.

Prior to the 3-week exercise training program, participants underwent two pre-training visits to determine baseline characteristics. Maximal oxygen consumption (VO_2max_), maximal aerobic workload (W_max_), and workload eliciting the lactate turnpoint (W_LT_) were assessed at the first visit. At a second visit 48 h later, plasma glucose and lactate metabolism were investigated at rest and during moderate-intensity exercise when fed glucose-fructose (metabolic evaluation). Participants were then separated into two parallel groups, and both groups performed 15 sessions of supervised moderate-intensity laboratory cycling on an ergometer (60 min each; 5 day·week^−1^) either with glucose-fructose drinks in GF or water in C. Finally, preliminary visits were repeated post-training (beginning 48–72 h after the last training session) to assess the effect of exercise training with glucose-fructose ingestion on metabolic response to these drinks, at rest and during exercise (Figure 1). To assume comparable glycogen concentrations between interventions, participants filled food diaries and were instructed to repeat dietary intake and physical activity patterns the 48 h prior to each visit.

### 2.3. Incremental Exercise-Testing

Overnight fasted participants performed an incremental test to exhaustion on a cycle ergometer (Ergoselect 100, Ergoline GmbH, Bitz, Germany) pre-training and post-training. Respiratory gas exchanges (SensorMedics Vmax; Sensormedics Corp., Yorba Linda, CA, USA) and heart-rate (Polar S810; Polar Electro Oy, Kempele, Finland) were continuously monitored throughout the test. Briefly, after a resting period of 5 min and a warm-up of 5 min at 40 W, ergometer workload was increased by 25 W every 3 min. As VCO_2_ exceeded VO_2_, ergometer workload was increased by 25 W every minute until volitional exhaustion. VO_2max_ and W_max_ were determined as previously described [13] and used to determine training intensities. Earlobe blood lactate concentration was measured at the end of each step (Lactate Pro, Arkray, Kyoto, Japan). Participants were then familiarized to the endurance capacity task (pre-training) or could leave the laboratory (post-training).

### 2.4. Metabolic Evaluations

Participants were instructed to remain sedentary, filled food diaries and had to avoid caffeine, alcohol and ^13^C-rich foods the 48 h before metabolic evaluations. Overnight-fasted participants reported to the metabolic unit at 0700 h and, after a void, were weighed and installed on a bed. One indwelling venous cannula was then inserted into an antecubital vein for blood sampling. This forearm was then constantly placed under a heating pad to open arteriovenous anastomoses, allowing for accurate determination of substrate exchanges in arterialized venous blood [18]. Another cannula was inserted into a vein of the opposite forearm for the infusion of stable isotopes tracers (Cambridge Isotope Lab., Andover, MT, USA). After background sampling at 0800 h (time = 0 min), a labelled-bicarbonate bolus (Na-H^13^CO_3_: 3.05 g) and primed continuous infusions of glucose ((6,6-^2^H_2_)-d-(+)-glucose; prime: 2 mg·kg^−1^; continuous: 0.02 mg·kg^−1^·min^−1^) and sodium lactate (Na-(3-^13^C_1_)-l-(+)-lactate; prime: 0.4 mg·kg^−1^; continuous: 0.02 mg·kg^−1^·min^−1^) were started. Infusion rates were tripled during exercise to account for increased substrate kinetics.

The metabolic evaluation consisted of a resting period (time = 0–90 min) in which participants remained in the supine position, followed by a continuous exercise session (time = 100–190 min) at 45% pre-training VO_2max_ (i.e., at the same workload pre-training and post-training). This intensity aimed to elicit the greatest effect of endurance-training on lactate metabolic clearance [19]. Glucose-fructose sweetened drinks (193 mL of a 9.8% glucose, 6.2% fructose drink flavored with 2% lemon juice and 1.17 g·L^−1^ NaCl) were provided at time = 0 then every 30 min at rest, and every 20 min during exercise. Blood and expired air samples were collected at time = 0, 30, 60, 75, and 90 min (rest), then 130, 145, 160, 175 and 190 min (exercise). Energy expenditure and substrate oxidation were measured by open-circuit indirect calorimetry (Quark RMR, Cosmed, Roma, Italia) in the last 30 min of rest and for 5 min intervals during exercise (SensorMedics Vmax; Sensormedics Corp, Yorba Linda, CA, USA). After 190 min, infusions were stopped and participants’ urine was collected to estimate protein oxidation.

Twenty-five minutes later, ergometer workload was set at 85% of the current VO_2max_ and participants were asked to cycle at 60 rpm until exhaustion to measure endurance capacity.

### 2.5. Training Intervention

Starting 48–72 h after the pre-training evaluation, participants entered a supervised endurance-training program of 1 session·d^−1^, 5 day·week^−1^ over 3 weeks. Each session consisted of 60 min cycling at a constant workload, with intensities set as 50% (sessions 1–3), 55% (sessions 4–6), 60% (sessions 7–9), and 65% (sessions 10–15) of pre-training VO_2max_. Experimental interventions differed by the drinks provided during sessions: the GF group ingested three 163 mL doses of glucose-fructose drinks provided −20, 0, and +20 min referred to exercise onset, while the C group correspondingly received water. Participants were instructed to have their last meal at least two hours before exercise onset. Earlobe blood lactate concentration was measured at 0, 30 and 60 min (Lactate Pro, Arkray, Japan).

### 2.6. Analytical Procedures

Arterialized venous blood samples were collected on lithium heparin for measurement of glucose, lactate, fructose, and tracers, with ethylenediaminetetraacetic acid (EDTA)-coated tubes for free fatty acids, triglycerides and insulin or with trasylol-EDTA for glucagon. Plasma was immediately separated by centrifugation (10 min; 2800× *g*; 4 °C), and aliquots were stored at −20 °C until analyzed. Plasma glucose, lactate, free fatty acids, and urinary nitrogen concentrations were determined using a semi-automated clinical chemistry analyzer (RX Monza, Randox Laboratories Ltd., Crumlin, UK). Insulin and glucagon concentrations were obtained by radioimmunoassay using commercial kits (Merck Millipore, Billerica, MA, USA).

Expired air ^13^CO_2_ isotopic enrichments were obtained by isotope-ratio mass spectrometry (IRMS) (SerCon Ltd., Crewe, UK), as previously described [13]. Gas chromatography-mass spectrometry (GCMS) (Hewlett-Packard Instruments, Palo Alto, CA, USA) was used to measure plasma fructose concentration [13] and plasma (^13^C_1_)lactate and (^2^H_2_)glucose isotopic enrichments [20,21]. Plasma (^13^C)glucose enrichments were measured by GC/C/IRMS [22] (Thermo Scientific, Bremen, Germany).

### 2.7. Calculations

Energy expenditure and substrate oxidation were calculated from respiratory gas exchanges using standard equations [23]. When exceeding VO_2_, VCO_2_ values were set as corresponding to VO_2_ to reflect aerobic metabolism, and ^13^CO_2_ isotopic enrichment was corrected for bicarbonate retention [24]. Rates of plasma glucose and lactate appearance, disposal, and metabolic clearance were calculated using Steele’s equations for non-steady state using a volume of distribution of 180 mL·kg^−1^ for both substrates [25]. Plasma lactate oxidation, non-oxidative lactate disposal (NOLD), and gluconeogenesis from lactate were calculated as:

(1) Lactate oxidation=lactate disposal·VCO2·CO213F·k·89.08 (mg·kg−1·min−1)

(2) $$\mathrm{NOLD}=\mathrm{lactate}\;\mathrm{disposal}-\mathrm{lactate}\;\mathrm{oxidation}\;(\mathrm{mg}\cdot {\mathrm{kg}}^{-1}\cdot \min^{-1})$$

(3) Gluconeogenesis from lactate=(C13)glucose·6·glucose appearance(C13)lactate (mg·kg−1·min−1)

where ^13^CO_2_ and (^13^C)glucose represent ^13^carbon isotopic enrichments in expired CO_2_ (atom% excess) and plasma glucose (atom% excess), (^13^C)lactate is (M+1)lactate isotopic enrichment (mol% excess), F is (3-^13^C_1_)lactate infusion rate, k is a correction factor for ^13^C losses in body pools during substrate oxidation [24] (rest: k = 0.8; exercise: k = 1.0), 89.08 is the molar weight of lactate and 6 is the number of mole of CO_2_ per mole of glucose. To minimize tracer assumptions, mean values of the last 30 min of rest (time = 60, 75 and 90 min) and exercise (time = 160, 175 and 190 min) are reported in figures (see results). Lactate turnpoint was obtained by the D-max method [18].

### 2.8. Statistics

Interventions allocation was determined by random generation of four-sequence blocks. A sample size of 16 participants was estimated (1-β: 90%; α = 0.05) to detect ≈15% difference in lactate clearance gain between GF and C. Normality and homoscedasticity were first checked visually, then using Shapiro-Wilk and Bartlett tests. Data were transformed in their square root when appropriate (plasma lactate, fructose, free fatty acids and insulin concentrations, carbohydrate and lipid oxidations, endurance capacity). Baseline values were compared using Student’s *t*-tests. Evolving data were analyzed using mixed-models, with training (T) and intervention (I) as fixed effects and random effects for participant-specific intercepts and slopes. The training and intervention interaction (T × I), baseline (B) and exercise (E) effects were included in models whenever improving goodness of fit. Paired contrasts were used to determine differences between pre-training vs. post-training (symbol: #) and rest vs. exercise periods (symbol: $), and unpaired contrasts to compare GF vs. C interventions (symbol: *). Analyses were run on R version 3.1.3 (R Foundation for Statistical Computing, Vienna, Austria). *p* < 0.05 was considered significant. Data are presented as mean ± SEM.

## 3. Results

### 3.1. Participants Characteristics and Training Effectiveness

**Figure 2:**
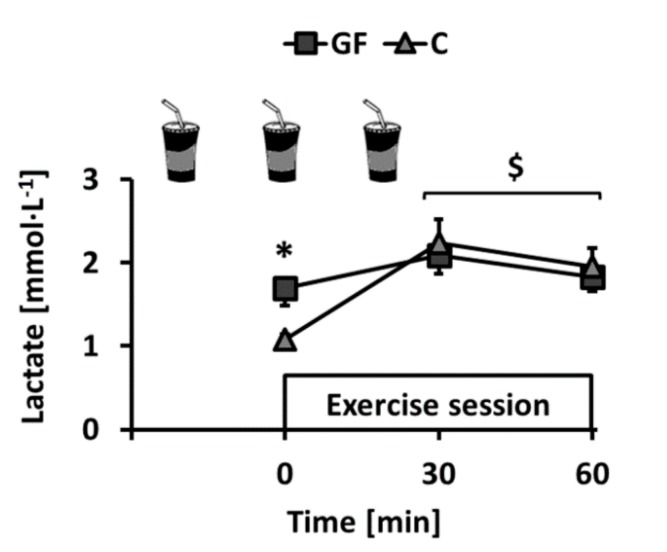
Changes over time of earlobe blood lactate concentration in GF and C groups during training sessions. GF received glucose-fructose drinks and C received water −20, 0, and +20 min relative to exercise onset. Effects of exercise (E) and intervention (I) were compared using a mixed-model analysis. Paired and unpaired contrasts were used to determine differences between rest and exercise (E effect: time = 0 min vs. time = 30–60 min: $: *p* < 0.01) and GF vs. C (I effect: *: *p* < 0.05). Mean ± SEM for *n* = 8 participants in all groups.

This study was completed between April 2012 and December 2014. All participants reported to have followed dietary instructions, completed every exercise session under investigators’ supervision and remained weight-stable throughout the experiments (T effect: *p* = 0.66; T × I effect: *p* = 0.54). Plasma lactate concentration was monitored immediately before and during training sessions. Ingestion of GF increased pre-session lactate as compared to C. Lactate was then increased by exercise but, interestingly, mean concentrations after 30 and 60 min exercise were not different (Figure 2: rest: *p* < 0.01; exercise: both *p* > 0.05).

Training was effective in both GF and C and increased VO_2max_, W_max_ and W_LT_ to similar extents (Table 1: all T effects: *p* < 0.01; T × I effects: *p* > 0.05). Consistent with improved conditioning, the fixed workload of the metabolic evaluation corresponded to lower relative exercise intensities post-training (GF and C, respectively 41% and 42% VO_2max_) than pre-training (45% VO_2max_ by design). Heart rate was decreased similarly in GF and C (all T effects: *p* < 0.01; all T × I effects: *p* > 0.05).

### 3.2. Metabolic Evaluation: Plasma Substrates and Hormones

**Figure 3:**
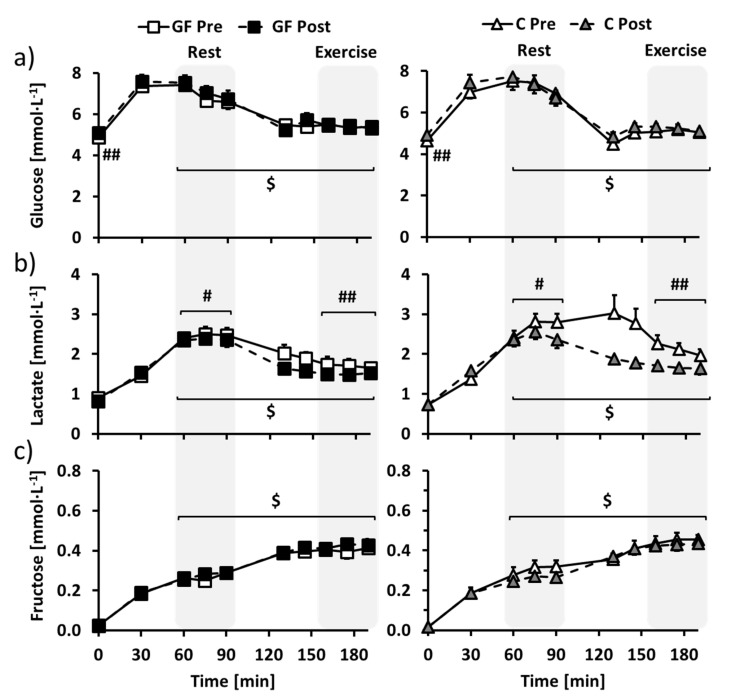
Changes over time of plasma (**a**) glucose, (**b**) lactate and (**c**) fructose concentrations in GF (**left**) and C (**right**) participants during metabolic evaluations. Glucose-fructose drinks were provided both at rest (time = 0–90 min) and during exercise (time = 100–190 min) in all tests. GF pre-training (GF Pre) and C pre-training (C Pre) is indicated in white, GF post-training (GF Post) in black and C post-training (C Post) in grey. Effects of exercise and interventions were compared using a mixed-model analysis. Paired contrasts were used for rest vs. exercise periods (E effect: $: *p* < 0.01) and pre- vs. post-training (T effect: #: *p* < 0.05; ##: *p* < 0.01). Dashed zones: Measures considered for tracer calculations. Mean ± SEM for *n* = 8 participants in all groups.

In overnight-fasted participants, training increased fasting glucose similarly in GF and C (Figure 3a: T effect: *p* < 0.01; T × I effect: *p* = 0.69), but did not affect plasma fructose, lactate, insulin and glucagon concentrations (Figure 3b,c, Figure S1a,b for insulin and glucagon: all effects: *p* > 0.05). Fasting free fatty acids concentration was decreased after training only in C (Figure S1c: T × I effect: *p* = 0.02).

With repeated ingestion of glucose-fructose drinks, glucose, lactate and fructose concentrations (Figure 3a–c) rapidly increased to stabilize in the last part of the resting period (time = 60–90 min). Plasma insulin followed the same time course, whereas glucagon decreased and free fatty acids were decreased below detectable values (Figure S1a–c; all T effect: *p* < 0.01). Regarding the effects of the interventions, plasma glucose was not affected by training (Figure 3a: T effect: *p* = 0.86; T × I effect: *p* = 0.56), while plasma lactate was decreased post-training compared to pre-training in both GF and C (Figure 3b: T effect: *p* = 0.02; T × I effect: *p* = 0.18). Plasma fructose tended to be increased in GF and decreased in C (Figure 3c: T effect: *p* = 0.39; T × I effect: *p* = 0.06) and other parameters were not altered by the interventions (Figure S1a–c: all T and T × I effects: *p* > 0.05).

As compared to rest, exercise then decreased glucose, lactate and insulin and increased fructose and glucagon concentrations (all E effects: *p* < 0.01) to new steady-state values in the last part of the exercise period (time = 160–190 min). However, free fatty acids concentrations remained below the detection limit (Figure S1c: E effect: *p* > 0.05). Plasma lactate was decreased post-training compared to pre-training in both GF and C (Figure 3b: T effect: *p* < 0.01; T × I effect: *p* = 0.13), while plasma glucose, fructose, free fatty acids, insulin and glucagon were unaffected by the interventions (Figure 3a,b, Figure S1a–c for insulin, glucagon and free fatty acids: all effects: *p* > 0.05).

### 3.3. Metabolic Evaluation: Isotopic Enrichments

**Figure 4:**
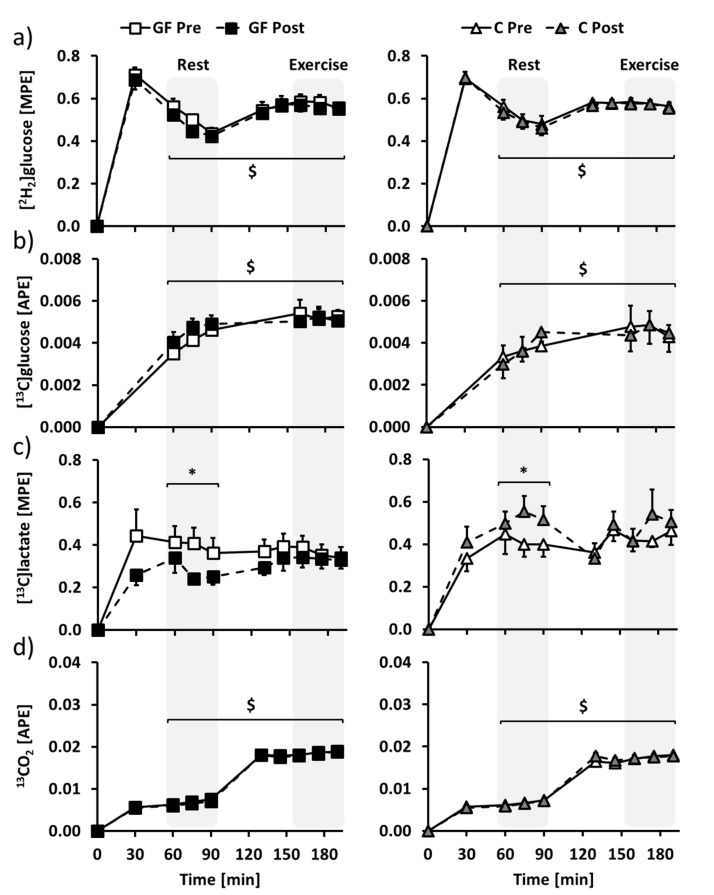
Changes over time of plasma (**a**) (^2^H_2_)glucose, (**b**) (^13^C)glucose, (**c**) (^13^C_1_)lactate and (**d**) expired air ^13^CO_2_ isotopic enrichments in GF (**left**) and C (**right**) participants during metabolic evaluations. Glucose-fructose drinks were provided in all tests, both during rest (time = 0–90 min) and exercise (time = 100–190 min) periods. GF pre-training (GF Pre) and C pre-training (C Pre) is indicated in white, GF post-training (GF Post) in black and C post-training (C Post) in grey. Effects of exercise and training interventions were compared using a mixed-model analysis. Paired contrasts were used for rest vs. exercise periods (E effect: $: *p* < 0.01) and training × interventions (T × I effect: *: *p* < 0.05). Dashed zones: Measures considered for tracer calculations. Mean ± SEM for *n* = 8 participants in all groups.

Glucose, lactate and CO_2_ isotopic enrichments (Figure 4) of the last 30 min of rest (time = 60–90 min) and exercise (time = 160–190 min) were selected to determine glucose and lactate metabolisms at rest and during exercise. Mean (^2^H_2_)glucose and (^13^C)glucose isotopic enrichments were increased from rest to exercise, but were not affected by both GF and C interventions (Figure 4a,b: E effects: *p* < 0.01; all T and T × I effects: *p* > 0.05). At rest, (^13^C)lactate isotopic enrichment was distinctly affected after GF and C (Figure 4c: T effect: *p* = 0.89; T × I effect: *p* = 0.02), and the difference was no longer significant during exercise (Figure 4c: T effect: *p* =0 .61; T × I effect: *p* = 0.18). ^13^CO_2_ isotopic enrichment was increased from rest to exercise, without being affected by GF or C (Figure 4d: E effect: *p* < 0.01; all T and T × I effects: *p* > 0.05).

### 3.4. Metabolic Evaluation: Glucose Metabolism

Summarized as mean values for selected periods of rest and exercise (Table 2), glucose appearance was similar in GF pre-training, GF post-training, C pre-training and C post-training at rest (T effect: *p* = 0.19; T × I effect: *p* = 0.99), then was increased to the same extent by exercise in all evaluations (E effect: *p* < 0.01; T effect: *p* = 0.71; T × I effect: *p* = 0.98). Glucose disposal was also similarly increased by exercise (rest: T effect: *p* = 0.19; T × I effect: *p* = 0.62; exercise: E effect: *p* < 0.01; T effect: *p* = 0.38; T × I effect: *p* = 0.90). Glucose clearance was also increased by exercise as compared to rest and remained constant after training in both GF and C (rest: T effect: *p* = 0.70; T × I effect: *p* = 0.74; exercise: E effect: *p* < 0.01; T effect: *p* = 0.78; T × I effect: *p* = 0.86).

### 3.5. Metabolic Evaluation: Lactate Metabolism

Distinctly from glucose metabolism, lactate metabolism was affected by GF and C interventions. Post-training, mean lactate appearance was increased in GF and decreased in C at rest (T effect: *p* = 0.56; T × I effect: *p* < 0.01). Lactate appearance was then significantly increased by exercise and, interestingly, the difference between GF and C interventions was no longer significant during exercise (E effect: *p* < 0.01; T effect: *p* = 0.68; T × I effect: *p* = 0.16). Lactate disposal was also differently affected by GF and C interventions at rest (T effect: *p* = 0.41; T × I effect: *p* < 0.01) and was increased by exercise (E effect: *p* < 0.01) during which differences between interventions were no longer significant (T effect: *p* = 0.91; T × I effect: *p* = 0.12). Lactate clearance followed the same trend at rest in which it was also differently affected by GF and C (T effect: *p* = 0.30; T × I effect: *p* = 0.02) and was then increased by exercise as compared to rest (E effect: *p* < 0.01). During exercise, lactate clearance was interestingly enhanced post-training compared to pre-training, but to similar extents in both GF and C (T effect: *p* < 0.01; T × I effect: *p* = 0.53).

### 3.6. Metabolic Evaluation: Lactate Disposal

The use of (^13^C_1_)lactate allowed to investigate several of its fates. Consistent with the effects of interventions on lactate metabolism, lactate oxidation (T effect: *p* = 0.60; T × I effect: *p* = 0.01), NOLD (T effect: *p* = 0.40; T × I effect: *p* < 0.01) and gluconeogenesis from lactate (T effect: *p* = 0.57; T × I effect: *p* = 0.01) measured during the resting period were all distinctly affected by GF and C interventions. Despite absolute values being affected by the interventions, lactate oxidation still represented a stable proportion of lactate disposal at rest (GF: 20.6 ± 1.0% to 18.6 ± 0.6% vs. C: 17.5 ± 1.7% to 17.7 ± 0.8%; T effect: *p* = 0.20; T × I effect: *p* = 0.52). In contrast, resting gluconeogenesis from lactate represented a larger part of glucose production after GF than after C (GF: 8.2 ± 1.5% to 11.9 ± 1.6% vs. C: 5.7 ± 1.2% to 5.0 ± 1.0%: T effect: *p* = 0.21; T × I effect: *p* = 0.01).

Compared to rest, exercise increased lactate oxidation, decreased NOLD and increased gluconeogenesis from lactate (all: E effects: *p* < 0.01). There were no more significant differences between GF and C interventions for absolute values of lactate oxidation (T effect: *p* = 0.74; T × I effect: *p* = 0.14), NOLD (T effect: *p* = 0.60; T × I effect: *p* = 0.77) and gluconeogenesis from lactate (T effect: *p* = 0.86; T × I effect: *p* = 0.99). Lactate oxidation represented a significantly higher part (and NOLD a lower part) of lactate disposal during exercise than at rest, yet without effect of GF or C interventions (lactate oxidation: GF: 86.3 ± 3.8% to 87.6 ± 4.9% vs. C: 86.8 ± 6.6% to 87.6 ± 6.6%; E effect: *p* < 0 .01; T effect: *p* = 0.99; T × I effect: *p* = 0.83). Similarly, no training effect was observed on fractional gluconeogenesis from lactate measured during exercise after both GF and C (GF: 10.7 ± 1.9% to 10.4 ± 1.6% vs. C: 7.0 ± 1.2% to 6.8 ± 1.5%; E effect: *p* = 0.06; T effect: *p* = 0.87; T × I effect: *p* = 0.97).

### 3.7. Metabolic Evaluation: Substrate Oxidation and Exercise Capacity

Carbohydrates provided most of the substrates to be oxidized throughout the evaluations (Table 3). Accordingly, lipid oxidation and protein oxidation (from urinary nitrogen collected during both periods) were low at rest and during exercise. In contrast, energy expenditure, total carbohydrate, lactate and other carbohydrate oxidations were all markedly increased during exercise as compared to the resting period (all E effects: *p* < 0.01).

As reported, at rest, lactate oxidation was increased post-training as compared to pre-training in GF, but not in C (T effect: *p* = 0.55; T × I effect: *p* = 0.01). Yet, this occurred within a stable total carbohydrate oxidation (T effect: *p* = 0.75; T × I effect: *p* = 0.61) and reflected an increasing proportion of carbohydrate oxidation coming from lactate oxidation after GF, but not after C (GF: 30 ± 6% to 38 ± 7% vs. C: 22 ± 4% to 15 ± 2%; T effect: *p* = 0.89; T × I effect: *p* < 0.01).

During exercise, there were no longer differences in lactate oxidation (T effect: *p* = 0.73; T × I effect: *p* = 0.11), total carbohydrate oxidation (T effect: *p* = 0.15; T × I effect: *p* = 0.71), or the fraction of total carbohydrate oxidation as lactate (GF: 35 ± 4% to 36 ± 4% vs. C: 26 ± 3% to 24 ± 3%; T effect: *p* = 0.92; T × I effect: *p* = 0.22) after interventions.

Finally, training with GF was hypothesized to specifically increase endurance capacity at 85% of current VO_2max_. Yet, time-to-exhaustion was similarly improved by GF and C interventions (Table 1: T effect: *p* < 0.01; T × I effect: *p* = 0.19).

## 4. Discussion

### 4.1. Efficiency of the Training Programs

Pre-training, participants’ VO_2max_ were in the middle range of normal values [26]. The exercise training programs were effective, and produced a +8% VO_2max_ increase similar to results from previous studies using a comparable training load [19,27]. Similar to other works comparing the effects of training with or without carbohydrate ingestion [27,28], no difference in performance gain was observed between GF and C.

### 4.2. Lactate Appearance and Energy Metabolism before Training

The initial metabolic evaluation involved the ingestion of repeated glucose-fructose drinks by both groups of participants at rest and during exercise. Since this visit was performed prior to intervention, results were expectedly similar in GF and C. In all participants, glucose-fructose ingestion increased blood lactate concentration compared to fasting values. Plasma lactate appearance, presumably from fructose in splanchnic tissues [29], but also resulting from glucose/glycogen degradation in various tissues including skeletal muscle, amounted to 3.6 mg·kg^−1^·min^−1^ in both GF and C. We did not measure fasting lactate appearance, but it was previously estimated as ≈1.4 mg·kg^−1^·min^−1^ in resting subjects [30]. This is consistent with GF being responsible for a substantial increase in lactate production. Our tracer approach does not allow the relative contributions of glucose and fructose to total lactate appearance to be estimated. Published human reports, however, indicate that intravenous fructose essentially stimulated splanchnic lactate release at rest [31] and during exercise [15], while animal studies suggest that glucose stimulated extra-splanchnic (presumably muscle) lactate production [32]. 

Gluconeogenesis from lactate was also minimal, most likely because it was inhibited by hyperinsulinemia induced by glucose ingestion [6]. Interestingly, lactate oxidation represented only ≈20% of lactate disposal, and the remaining ≈80% was metabolized non-oxidatively. Since there is no substantial lactate and glucose stores in the human body, this most likely corresponded to liver and/or muscle glycogen synthesis. In support of this hypothesis, we recently reported that post-exercise muscle glycogen resynthesis was quantitatively similar when subjects were fed glucose or fructose, and that lactate concentrations elicited by fructose ingestion were positively correlated with muscle glycogen resynthesis [33].

During exercise, lactate appearance increased to ≈10 mg·kg^−1^·min^−1^, of which ≈30% may have derived from fructose contribution, according to a previous study using comparable glucose-fructose drinks [13]. Relative to resting conditions, plasma lactate concentrations decreased, reflecting the effect of exercise to increase muscle lactate uptake and hence lactate clearance [30]. In addition, exercise directed ≈90% of lactate appearance toward oxidation, representing a much larger fraction than at rest. This is consistent with previous investigations of lactate metabolism in unfed individuals [10,11,34], confirming that lactate disposal between oxidative and non-oxidative fates is largely dictated by metabolic rate [7], also with glucose-fructose ingestion. We postulate that the transfer of lactate from splanchnic organs to working muscle after glucose-fructose ingestion (i.e., “reverse Cori cycle”) is the result of two simultaneous processes: first, an increase of splanchnic lactate production pushed by fructose lacticogenesis increasing intrasplanchnic lactate concentration, and thus lactate efflux; second, an increased muscle lactate uptake pulled by low intramuscular lactate concentration due to continuous lactate removal toward oxidation.

### 4.3. Evolution of Plasma Lactate Concentration during Training Sessions

There was no detailed metabolic evaluation during training sessions. However, plasma lactate concentration was measured throughout the training program at rest and during exercise. At rest, plasma lactate concentration was higher in the GF group than in the C group, reflecting the well-known increase in plasma lactate induced by fructose ingestion [29,35]. During exercise, interestingly, plasma lactate concentration was similar in both groups, suggesting that the effect of glucose-fructose drinks was minor compared to that of exercise per se [30].

### 4.4. Lactate Appearance and Disposal after Training

All participants returned at the end of the training program for a second evaluation with glucose-fructose ingestion at rest and during exercise. The resting period revealed differences after GF and C interventions. Interestingly, unlike C that induced a decrease in lactate metabolism, confirming previous reports [19,34,36], GF differed by increasing lactate appearance. This may derive from an enhanced capacity to digest, absorb and metabolize fructose [29,37] or reflect the increased lactate appearance observed in fasted individuals after a few days of fructose exposure [38]. The mechanisms of such adaptations remain to be elucidated. Lactate clearance was increased in GF only, while lactate oxidative and non-oxidative disposal remained remarkably stable after training. Interestingly, resting lactate metabolism was increased by GF along with an unchanged glucose metabolism and carbohydrate oxidation, consistent with considerations that lactate may be preferred over other substrates for energy or glucose production [7].

In contrast to the resting period, lactate appearance measured during exercise was unaltered by training. Lactate clearance, however, increased by ≈20% in both GF and C, and plasma lactate concentration decreased. This can be attributed to an enhanced expression of muscle lactate transporters and lactate metabolizing enzymes [8]. However, and contrary to our hypothesis, training with glucose-fructose did not potentiate the effects of training alone. This observation is in line with the fact that lactate concentration was similar during training sessions with or without glucose-fructose drinks, and suggests that any additional effect of glucose-fructose ingestion during exercise was minimal compared to the effects of exercise training on lactate metabolism.

### 4.5. Limitations

First, glucose-fructose drinks were used as a tool to change lactate metabolism through effects on lactate concentration. While measuring blood lactate concentration during training sessions, our experimental protocol did not allow to assess intrasplanchnic and intramuscular lactate concentrations. Second, participants’ diet was not entirely controlled during the training period and we cannot ascertain how glucose-fructose drinks modified total sugars intake in GF and C interventions. Third, our choice of tracers did not allow endogenous glucose appearance to be distinguished from exogenous glucose appearance, nor lactate appearance from fructose and from glucose-lactate shuttles. Four, the small sample size may have prevented the detection of still meaningful differences.

## 5. Conclusions

Ingestion of glucose-fructose drinks increase lactate appearance, metabolism and plasma lactate concentration above fasting values. This lactate is then mainly metabolized non-oxidatively at rest (presumably ending up in glycogen stores [33]) and oxidatively during exercise [13]. After having completed the 3-week training program, the ingestion at rest of glucose-fructose drinks increased lactate appearance and oxidation more in subjects who had received glucose-fructose during sessions than in those who had received water. This suggests that repeated glucose-fructose ingestion during training upregulated fructose absorption and splanchnic lacticogenesis from fructose. During exercise, however, lactate appearance and oxidation remained unchanged compared to pre-training conditions, indicating that neither training nor glucose-fructose consumption had a major impact on splanchnic lacticogenesis from fructose.

## Figures and Tables

**Table 1:** Participants’ body weight and performance parameters.

	GF Pre	GF Post	C Pre	C Post
Body weight (kg)	73.7 ± 3.0	73.6 ± 2.8	72.9 ± 2.9	73.1 ± 3.0
VO_2max_ (mL·kg^−1^·min^−1^)	44.3 ± 2.3	48.4 ± 2.0 #	46.4 ± 2.2	49.4 ± 2.1 #
W_max_ (W)	249 ± 20	281 ± 19 #	249 ± 16	287 ± 21 #
W_LT_ (W)	156 ± 15	180 ± 15 #	156 ± 12	181 ± 14 #
Endurance capacity (s)	663 ± 110	1134 ± 163 #	687 ± 177	1455 ± 293 #

Changes of participants’ body weight and performance parameters. Baseline values were compared using an unpaired Student’s *t*-test. Effects of training interventions were compared using a mixed-model analysis. Paired contrasts were used to determine differences between pre- vs. post-training (T effect: #: *p* < 0.01). GF: glucose-fructose intervention; C: control intervention; Pre: pre-training; Post: post-training; VO_2max_: maximal oxygen consumption; W_max_: maximal workload; W_LT_: workload at lactate turnpoint. SEM: standard error of the mean; Mean ± SEM for *n* = 8 participants in all groups.

**Table 2:** Glucose and lactate fluxes in the resting and exercise periods

		GF Pre	GF Post	C Pre	C Post
Glucose appearance (mg·kg^−1^·min^−1^)	Rest	5.6 ± 0.3	5.8 ± 0.2	5.1 ± 0.4	5.4 ± 0.4
	Exercise	10.8 ± 0.6 $	10.9 ± 0.5 $	10.5 ± 0.3 $	10.7 ± 0.3 $
Lactate (mg·kg^−1^·min^−1^)	Rest	0.5 ± 0.1	0.7 ± 0.1 *	0.3 ± 0.0	0.3 ± 0.1 *
	Exercise	1.2 ± 0.2 $	1.1 ± 0.2 $	0.7 ± 0.1 $	0.7 ± 0.2 $
Other (mg·kg^−1^·min^−1^)	Rest	5.1 ± 0.3	5.1 ± 0.2	4.8 ± 0.5	5.1 ± 0.4
	Exercise	9.6 ± 0.6 $	9.8 ± 0.6 $	9.8 ± 0.3 $	9.9 ± 0.3 $
Glucose disposal (mg·kg^−1^·min^−1^)	Rest	6.2 ± 0.5	6.4 ± 0.4	5.5 ± 0.6	6.0 ± 0.5
	Exercise	10.8 ± 0.7 $	11.2 ± 0.5 $	10.5 ± 0.3 $	10.8 ± 0.3 $
Glucose clearance (mL·kg^−1^·min^−1^)	Rest	5.0 ± 0.5	5.0 ± 0.4	4.4 ± 0.6	4.7 ± 0.4
	Exercise	11.2 ± 0.9 $	11.5 ± 0.7 $	11.5 ± 0.6 $	11.5 ± 0.3 $
Lactate appearance (mg·kg^−1^·min^−1^)	Rest	3.6 ± 0.5	5.2 ± 0.7 **	3.6 ± 0.4	2.6 ± 0.5 **
	Exercise	11.2 ± 1.4 $	12.1 ± 1.5 $	8.8 ± 0.7 $	8.3 ± 0.9 $
Lactate disposal (mg·kg^−1^·min^−1^)	Rest	3.4 ± 0.5	5.0 ± 0.7 **	3.2 ± 0.4	2.5 ± 0.4 **
	Exercise	11.3 ± 1.4 $	12.1 ± 1.5 $	9.1 ± 0.7 $	8.4 ± 1.0 $
Oxidation (mg·kg^−1^·min^−1^)	Rest	0.7 ± 0.1	0.9 ± 0.1 *	0.6 ± 0.1	0.4 ± 0.1 *
	Exercise	9.7 ± 1.4 $	10.6 ± 1.7 $	7.9 ± 1.0 $	7.3 ± 1.1 $
NOLD (mg·kg^−1^·min^−1^)	Rest	2.7 ± 0.4	4.1 ± 0.6 **	2.7 ± 0.4	2.0 ± 0.4 **
	Exercise	1.5 ± 0.3 $	1.5 ± 0.4 $	1.2 ± 0.6 $	1.0 ± 0.5 $
Lactate clearance (mL·kg^−1^·min^−1^)	Rest	17.8 ± 3.0	26.9 ± 4.9 *	16.0 ± 2.6	13.1 ± 2.6 *
	Exercise	75.5 ± 8.7 $	91.0 ± 9.6 $#	47.6 ± 4.7 $	57.6 ± 7.0 $#

Mean values during rest (time = 60–90 min) and exercise (time = 160–190 min) periods of metabolic evaluations performed pre-training (Pre) and post-training (Post). Effects of exercise and training interventions were compared using a mixed-model analysis. Paired and unpaired contrasts were used for rest vs. exercise periods (E effect: $: *p* < 0.01), pre- vs. post-training (T effect: #: *p* < 0.01) and training × interventions (T × I effect: *: *p* < 0.05; **: *p* < 0.01). NOLD: non-oxidative lactate disposal. Mean ± SEM for *n* = 8 participants in all groups.

**Table 3:** Fuel Selection in the Resting and Exercise Periods of Metabolic Evaluations

		GF Pre	GF Post	C Pre	C Post
Energy expenditure (kcal·min^−1^)	Rest	1.5 ± 0.1	1.5 ± 0.0	1.4 ± 0.1	1.4 ± 0.1
	Exercise	8.3 ± 0.5 $	8.4 ± 0.6 $	8.9 ± 0.5 $	9.0 ± 0.6 $
Protein (mg·kg^−1^·min^−1^)	Both	0.8 ± 0.0	0.8 ± 0.1	0.8 ± 0.1	0.8 ± 0.1
Lipid (mg·kg^−1^·min^−1^)	Rest	0.8 ± 0.1	0.8 ± 0.1	0.6 ± 0.1	0.6 ± 0.2
	Exercise	0.8 ± 0.3	0.5 ± 0.3	0.4 ± 0.2	0.8 ± 0.3
Carbohydrate (mg·kg^−1^·min^−1^)	Rest	2.8 ± 0.4	2.8 ± 0.3	2.9 ± 0.4	2.8 ± 0.4
	Exercise	27.6 ± 2.4 $	28.5 ± 2.6 $	30.6 ± 1.7 $	30.0 ± 1.7 $
Lactate (mg·kg^−1^·min^−1^)	Rest	0.7 ± 0.1	0.9 ± 0.1 *	0.6 ± 0.1	0.4 ± 0.1 *
	Exercise	9.7 ± 1.4 $	10.6 ± 1.7 $	7.9 ± 1.0 $	7.3 ± 1.1 $
Other (mg·kg^−1^·min^−1^)	Rest	2.1 ± 0.4	1.8 ± 0.4	2.4 ± 0.5	2.3 ± 0.4
	Exercise	17.8 ± 1.5 $	17.9 ± 1.4 $	22.7 ± 1.4 $	22.7 ± 1.8 $

Mean values during rest (time = 60–90 min) and exercise (time = 160–190 min) periods of metabolic evaluations performed pre-training (Pre) and post-training (Post). Effects of exercise and training interventions were compared using a mixed-model analysis. Paired and unpaired contrasts were used for rest vs. exercise periods (E effect: $: *p* < 0.01) and training × interventions (T × I effect: *: *p* < 0.05). Mean ± SEM for *n* = 8 participants in all groups.

## Supplementary Materials

The following are available online at www.mdpi.com/2072-6643/9/4/411/s1, Figure S1: Changes over time of plasma insulin, glucagon free fatty acids concentrations during metabolic evaluations.

## Abbreviations

C	Control intervention in which plain water was provided during training sessions
GF	Intervention in which glucose-fructose drinks were provided during training sessions
VO_2max_	Maximal oxygen consumption during incremental tests
W_max_	Maximal workload during incremental tests
W_LT_	Workload eliciting lactate turnpoint during incremental tests
VO_2_	Oxygen consumption
VCO_2_	Carbon dioxide production
Na-H^13^CO_3_	Sodium bicarbonate with its carbon being a carbon-13
NOLD	Non-oxidative lactate disposal
(6,6-^2^H_2_)-d-(+)-glucose	d-(+)-glucose deuterated twice on 6th carbon
(3-^13^C_1_)-l-(+)-lactate	l-(+)-lactate with its 3rd carbon being a carbon-13
NaCl	Sodium chloride
EDTA	Ethylenediaminetetraacetic acid
