# Supplementary material for: Endurance Training with or without Glucose-Fructose Ingestion: Effects on Lactate Metabolism Assessed in a Randomized Clinical Trial on Sedentary Men
Source: Nutrients. 2017 Apr 20;9(4):411. doi: 10.3390/nu9040411 (PMC5409750; doi:10.3390/nu9040411)
Supplement: Supplementary file 1 [file nutrients-09-00411-s001.pdf]

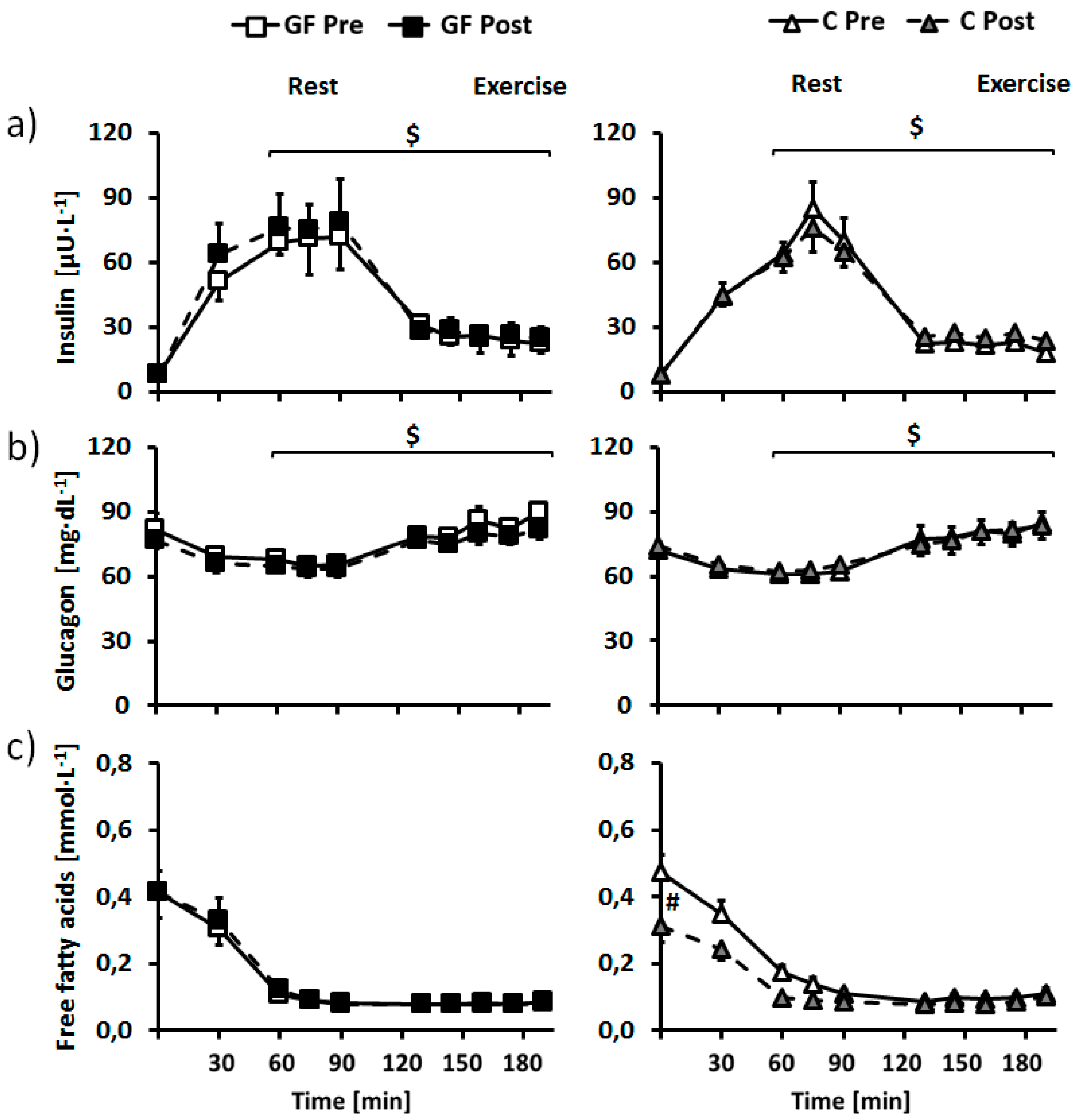

Figure S1. Changes over time of plasma (**a**) insulin, (**b**) glucagon, and (**c**) free fatty acids concentrations in GF (**left**) and C (**right**) participants during metabolic evaluations. Glucose-fructose drinks were provided both at rest (time = 0–90 min) and during exercise (time = 100–190 min) in all tests. GF pre-training (GF Pre) and C pre-training (C Pre) is indicated in white, GF post-training (GF Post) in black, and C post-training (C Post) in grey. Effects of exercise and interventions were compared using a mixed-model analysis. Paired contrasts were used for rest vs. exercise periods (E effect: $: $p < 0.01$), and pre- vs. post-training (T effect: #: $p < 0.01$). Mean ± SEM for $n = 8$ participants in all groups.
